# An Exploratory Study on the Influence of Psychopathological Risk and Impulsivity on BMI and Perceived Quality of Life in Obese Patients

**DOI:** 10.3390/nu9050431

**Published:** 2017-04-26

**Authors:** Renata Tambelli, Luca Cerniglia, Silvia Cimino, Giulia Ballarotto, Marinella Paciello, Carla Lubrano, Serena Marchitelli, Lucio Gnessi, Andrea Lenzi

**Affiliations:** 1 Department of Dynamic and Clinical Psychology, Sapienza University of Rome, 00185 Rome, Italy; renata.tambelli@uniroma1.it (R.T.); silvia.cimino@uniroma1.it (S.C.); giulia.ballarotto@uniroma1.it (G.B.); 2 Faculty of Psychology, International Telematic University UNINETTUNO, 00100 Rome, Italy; m.paciello@uninettunouniversity.net; 3 Department of Experimental Medicine-Medical Physiopathology, Food Science and Endocrinology Section, Sapienza University of Rome, 00185 Rome, Italy; carla.lubrano@uniroma1.it (C.L.); serena.marchitelli@uniroma1.it (S.M.) lucio.gnessi@uniroma1.it (L.G.); andrea.lenzi@uniroma1.it (A.L.)

**Keywords:** obesity, perceived quality of life, psychopathological risk, impulsivity

## Abstract

The present study aimed to assess the psychological profiles of adult male and female obese patients, as well as to verify the possible influence of their psychopathological risk and impulsivity on their body mass index (BMI) and perceived quality of life. A total of 64 obese subjects accessing a center for care of their obesity were assessed through anthropometric and psychometric measurements. All anthropometric measures in men were higher than in women, while in turn, women showed higher psychopathological symptoms. Furthermore, the symptoms of somatization and psychoticism were predictors for a higher BMI in men, but there was no effect of psychopathological symptoms on the perceived quality of life (QoL) of male subjects. Moreover, in women, somatization and attentional impulsivity were predictors for a higher BMI, whereas no correlation was found between their psychopathological risk and perceived QoL. The results of regression analysis underlined that somatization is a “core” psychopathological symptom in obese subjects regardless of their sex, which is a potential predictor for a higher BMI. The psychological difficulties of the subjects had no effect on their perceived QoL, suggesting that they find it difficult to reflect on the impact that obesity has on their life.

## 1. Introduction

According to the World Health Organization [1], more than 600 million people worldwide have a body mass index (BMI) greater than or equal to 30 kg/m^2^, with obesity having reached the proportions of a global pandemic. Internationally, the prevalence of being overweight and obesity combined is approximately 37% for men and 38% for women, with the number of overweight and obese individuals having increased from 921 million in 1980 to 2.1 billion in 2013 [2]. These epidemiological studies have posited that, if not effectively monitored and treated, obesity may become a standard condition for subsequent generations and cause a decrease in life expectancy over the next few decades. Newby and Tucker [3] highlighted the importance of identifying organic and psychological characteristics in patients with disordered eating to implement personally tailored intervention plans to reduce weight. In line with this study [3], previous literature has mostly addressed the association of metabolic syndrome (MetS), central adiposity, and the level of obesity (measured by BMI) with altered psychological functioning. It has therefore been posited that obese subjects who have a high BMI and MetS also suffer from higher psychopathological symptoms [4]. These associations have been recognized in both men and women. However, several authors suggested that it is important to consider the differences between sexes in the study of psychopathological risk in obese patients [5,6]. In fact, obese women usually report higher psychopathological problems than obese men, and women show specific constellations of symptoms [7]. This specificity also applies to the reasons that men and women report for their overeating or binge episodes. Men usually overeat due to environmental reasons and/or in response to subjective sensations of hunger, whereas this behavior occurs in women more frequently as a result of stressful experiences or negative emotions [8].

Although the associations between BMI and psychopathological problems have been widely explored, most potential interventions that aim to diminish excessive adiposity are based on augmented physical activity and caloric restriction. These methods show limited success (especially among women) [9], since individuals who do experience successful weight loss fail to maintain it over time [10]. According to several authors, this is because these interventions do not specifically address the underlying psychological and neurobiological factors that account for dysregulated eating behaviors [11], either as associated factors or as predictors of obesity itself. However, recent findings in neurobiology have demonstrated that at least some types of maladaptive organic conditions (such as being overweight and obesity in adults) share common underlying mechanisms with psychopathology. For instance, it has been theorized that hyperphagia—or chronic overeating, which is a key behavioral element of obesity [12]—is produced (and reinforced) by an alteration of the reward circuitry in the brain, which is also responsible for impulsivity and impaired stress in addition to affecting regulation [13]. Clinical and social psychology [14,15] has gone even further in proposing a causal effect of psychopathological symptoms (e.g., somatization, anxiety, and depression) on overeating and obesity, suggesting that dysregulated eating behaviors are used to cope with elevated levels of mental distress (emotional eating) [16]. This mechanism has been proposed to be more active in impulsive subjects, who frequently report impaired social and relational adjustment [17]. 

Disentangling the direction of the relationship that links psychopathological risk and obesity might inform both treatment programs and assessment protocols. This highlights the importance of specifically screening for mental distress in obese populations not merely as an associated factor that supports intervention, but as a key element to work on clinically, in order to promote effective and long-lasting weight loss. Research has demonstrated through longitudinal studies that psychopathology in childhood and adolescence is a robust predictor of obesity in adulthood [18,19]. Nevertheless, the role of mental health in the actual fostering of obesity in adults has been scarcely addressed, due to the limited power of cross-sectional studies in demonstrating causal links between variables. However, some authors have recently posited that even cross-sectional research can offer useful information on causal relationships in clinical settings, provided that it is based in a robust methodology [20,21]. 

On the basis of the above considerations, the present study primarily aimed to assess:

• Anthropometric parameters (i.e., weight, height, waist circumference, and BMI) in obese male and female adults.
• Their psychological profiles.

Our secondary aim was to verify:

• The possible influence of the psychopathological risk and impulsivity of obese men and women on their BMI and perceived quality of life (QoL).

Although the associations between obesity, perceived QoL, and psychopathology have been widely addressed (even in large samples), most studies considered only depression and anxiety, whereas this study included a broad series of psychopathological symptoms. Another study also did so [22], but to the best of our knowledge, no study has focused on the same objectives in an Italian context.

## 2. Materials and Methods 

Study participants were enrolled from the patients accessing the Casco High Specialization Center for the Care of Obesity at the University Hospital “Policlinico Umberto I”, Sapienza University of Rome, Italy, from March 2015 to December 2016. All patients accessing the Casco Center that were selected as subjects agreed to participate in the study, and no attrition occurred. The Casco Center’s aim is to assess subjects through endocrinological, cardiological, metabolic, nutritional, and psychological screenings in the form of day visits (all the screening procedures for the subjects of this study were performed on different and subsequent days in a randomized order, so as not to affect results). Based on the diagnosis, patients underwent a nutritional and a psychological intervention (and/or surgical treatment if needed). Inclusion criteria for the subjects of the present study were being aged between 18 and 65 years of age, a BMI greater than or equal to 30 kg/m^2^, and being Caucasian Italian. The exclusion criteria were: any psychiatric disorder; any reported malignant disease in the last five years; any reported or diagnosed inflammatory or autoimmune disease; corticosteroids for systemic use; any medications potentially affecting body weight, body composition, or mental status; participation in a weight loss program in the last three months; renal failure; heart failure; history of viral or autoimmune liver disease or any other chronic liver disease; or excessive alcohol intake (≥140 g/week for men or 70 g/week for women). Although we excluded all patients who were referred for any psychiatric disorder from this study, we performed a screening for binge eating disorder (BED) using the Binge Eating Scale (BES) [23] to avoid possible omissions, as reported in the preliminary analyses paragraph. None of the subjects in our sample were candidates for bariatric surgery.

The sample was comprised of 64 obese subjects (23 males and 41 females) who lived in Central Italy. Of the sample, 42.5% were married, 9.3% were divorced, 38.7% were single, and 4.2% were widows/widowers. The overall sample had an average socioeconomic status (SES) [24]. The study was conducted in accordance with the Declaration of Helsinki. Before the start of the study, the protocol was approved by the Ethics Committee of Sapienza, University of Rome (ethical approval code: n. 3920-2015). All participants signed a written informed consent.

*Psychopathological symptoms*: The Symptom Checklist (SCL-90-R) is a 90-item self-report symptom inventory designed to measure psychological symptoms and psychological distress [25]. It is scored and interpreted in terms of nine primary subscales (somatization, obsessive-compulsivity, interpersonal sensitivity, depression, anxiety, hostility, phobic anxiety, paranoid ideation, and psychoticism) and three global indices of distress (global severity index, positive symptom distress index, and positive symptom total). The SCL-90-R is rated on a Likert scale of 0 (not at all) to 4 (extremely), and asks participants to report whether they have suffered in the past week from: headaches (somatization scale), trouble remembering things (obsessive-compulsivity scale), feeling critical of others (interpersonal sensitivity scale), blaming oneself for things (depression scale), feeling fearful (anxiety scale), irritability (hostility scale), feeling of fear (phobic anxiety scale), feeling watched or talked about by others (paranoid ideation scale), and the idea that something is wrong with one’s mind (psychoticism scale). In general, higher scores indicate higher psychological distress and greater psychological symptoms. Prunas et al. [26] demonstrated the satisfactory internal consistency of the Italian version of the SCL-90-R in adolescents and adults (α coefficient, 0.70–0.96). We used the Italian version of the SCL-90-R [25].

*Impulsivity*: The Italian version of the Barratt Impulsiveness Scale (BIS-11; [27]) is a self-report questionnaire designed to assess impulsivity and its components. The 30 items in this questionnaire are measured on a four-point ordinal scale (from rarely/never to almost always/always). A review of studies using the BIS-11 on adult samples reported good psychometric properties [28]. The scale showed good internal consistency (α = 0.79), two-month test–retest reliability (α = 0.89), and criterion-related validity in addition to demonstrating satisfactory clinical usefulness. Moreover, an exploratory principal components analysis replicated the six first-order factors and three second-order factors of the original version (albeit with different item loadings).

*Quality of life (QoL)*: The Obesity-Related Well-Being (ORWELL) scale [29] was developed to examine the occurrence of physical and psychosocial symptoms as well as the subjective relevance of these symptoms in obese patients. The ORWELL 97 consists of 18 items on a four-point Likert scale. Items are scored by multiplying the impairment for a given item by the self-rated importance of each item. Therefore, if there is a high impairment in “sexual functioning” but the subject scores the relevance as “0—not important at all”, they will not show an impaired QoL based on that item. The measure has shown good internal consistency (α = 0.83). A score in the ORWELL-97 questionnaire ≥70—corresponding to the 75th percentile of the population—was considered indicative of a clinically significant burden of obesity in terms of QoL.

### Statistical Analysis 

Data were analyzed using the IBM SPSS statistics software, version 23. Both qualitative and quantitative analyses were performed on the data. A power analysis was conducted on the basis of Cohen’s indications [30] with the α set at 0.05, and a power of 0.789 was obtained with a large effect size (*f*^2^ = 0.48). Missing data for all applicable analyses were corrected using multiple imputations in SPSS. 

The qualitative analyses were run separately for each gender using descriptive statistics (reliability of the measures, frequencies, mean scores, and percentages). To verify whether subjects’ psychopathological risk and levels of impulsivity had an impact on their BMI and perceptions of QoL, linear regression analyses were conducted separately for male and female subjects. Methodological issues may contribute to mixed findings for the relationship between psychopathology and obesity in cross-sectional studies. For this reason, we followed the recommendations of Di Pietro et al. [31] and tested for the impact of confounding variables, such as smoking, alcohol use, concurrent medical illness, and SES. All presented results were adjusted for these confounding variables.

## 3. Results

### 3.1. Preliminary Analyses

Sixty-four obese subjects were included on the basis of the above criteria (23 men and 41 women; mean age: 44.9 ± 12.7 vs. 46.1 ± 14.6 years, *p* = 0.74; mean BMI: 42 ± 8.2 vs. 37.5 ± 6.9 kg/m^2^, *p* < 0.05).

Men had an average higher waist circumference than women (133.3 ± 15.3 vs. 116.9 ± 14.8 cm; *p* < 0.001). In our sample, 49.3% of subjects had MetS (25.7% men vs. 74.3% women). To conduct a preliminary test for the possible effect of the presence of MetS on the SCL-90-R subscales, all BIS subscales, BMI, and QoL scores, an ANOVA was conducted on all total scores. The results showed no significant differences in these scores in subjects with or without MetS. 

In acknowledgment of the studies of several authors who demonstrated the association as well as the predictive and/or mediating and moderating effect of BED on BMI and psychopathology (for a particularly informative paper, see Reference [32]), all our subjects were administered the BES as a part of the clinical protocol. Twenty-one subjects out of 64 exceeded the cutoff of 17 for BED. As a result, we replicated Paterson’s method to verify the effect of BED on BMI and psychopathology. However, we did not find the expected effect. In light of the previous literature demonstrating the clinical usefulness of results showing associations between psychopathology and BMI without considering possible comorbid eating disorders [33,34], we decided not to take BED into account in our methodology, focusing instead on the effect of psychopathological risk on BMI and QoL.

### 3.2. Psychological Profiles of Obese Subjects

To fulfill our first objective, we ran descriptive statistics separately for male and female subjects. The psychological profiles and anthropometric measurements of the study participants are described in Table 1. Two interesting findings are evident: first, all anthropometric measures in men were higher than in women; second, women showed higher psychopathological scores in all subdimensions of the SCL-90 except hostility, paranoid ideation, and psychoticism. Consistent with this result, there were significantly higher scores for the Global Severity Index in women than in men. No significant difference was found between the scores for men and women on the BIS and ORWELL 97.

### 3.3. Influence of Psychopathological Risk and Impulsivity on Body Mass Index (BMI) and Quality of Life

To fulfill our second objective and verify whether subjects’ psychopathological symptoms and levels of impulsivity had an impact on their BMI and perceived QoL, regressions analyses were conducted separately for male and female subjects. The results are shown in Table 2 and Table 3. In men, somatization and psychoticism predicted BMI scores (*p* < 0.05), but no effect of psychopathological symptoms was found using the ORWELL 97. 

In women, somatization and attentional impulsivity had an effect on BMI (*p* < 0.05), whereas no correlation was found between the SCL-90 and BIS variables on QoL scores.

## 4. Discussion

The present study aimed to assess the psychological profiles of adult male and female obese patients, as well as to verify the possible influence of psychopathological risk and impulsivity on BMI and perceived QoL. Cleoburry and Tapper [35] recommended considering sex differences when studying overweight and obese patients and their related psychological profiles, because it has been suggested that men and women may overeat (and therefore be obese) for different reasons. Specifically, men usually report more environmental, bodily sensation, and hunger reasons for overeating, whereas women report more ruminant thoughts, cognitive, and social reasons [36], as they have higher levels of emotional eating (eating in response to stress and other negative emotions) [37,38]. In fact, our results showed higher psychopathological scores in women in all subdimensions of the SCL-90, except for hostility, paranoid ideation, and psychoticism (which were not significantly different from the scores in men). This is an interesting finding, considering that (as noted above) all results are adjusted for potentially confounding variables (SES, alcohol or drug use, etc.) and that this sample did not report other concurrent risk factors (e.g., physical illness, psychiatric diagnosis). Moreover, previous studies have found that obese women were more prone to having psychological problems compared to obese men [39], yet this sample did not demonstrate such a notable difference. No significant difference was found between the scores for men and women in terms of impulsivity and perceived QoL. Moreover, while the psychopathological symptoms of women in many cases exceeded the clinical cutoffs (or were high; see Table 1), the impulsivity and perceived QoL scores were notably below the clinical cutoffs in both men and women (BIS cutoff ≥60; ORWELL 97 cutoff ≥70). We speculate that the low scores on the BIS and ORWELL 97 should be considered as a single, more complex factor. In fact, some authors have posited that impulsivity is a key variable accounting for dysregulated eating in obese subjects [40,41]. Nevertheless, other studies have suggested that the perception of a good quality of social and relational functioning (i.e., QoL) may act as a moderator of impulsivity in normal-weight and obese individuals alike [42,43,44]. On the other hand, especially in the case of women, we have to consider the indications from their self-reported psychopathological symptoms. How is it possible that women reporting high symptoms of somatization, obsession-compulsivity, and depression perceive a good QoL? We hypothesize that the capacity of these subjects to reflect on and self-represent their condition might be impaired by poor mental health and particularly by low reflective function and self-monitoring where body image is concerned [45]. In fact, it has been suggested that individuals with disordered eating and/or eating disorders show an impaired cognitive processing, as they typically underestimate and denigrate the negative impact of their condition on their QoL [46,47]. In other words, female obese subjects could effectively report psychological suffering (hence, they report high scores on the SCL-90) while conversely being unable to recognize the impact of obesity on their social and relational adjustment. 

With regards to the possible influence of psychopathological symptoms and impulsivity on subjects’ BMI and perceived QoL, we found that somatization and psychoticism in men and somatization and attentional impulsivity in women predicted BMI scores. However, in a cross-sectional study (see the introduction section), these results might constitute a pivotal step forward after several seminal studies that have demonstrated the association (i.e., a non-causal link) between psychopathological symptoms and obesity in adults [48,49]. This association has been supported by eminent biological studies that have suggested that alterations in brain structure and in the functionality of specific neurobiological circuitry may result in a predisposition to both obesity and certain forms of psychopathology [50,51]. Recent findings showed common brain monoamines and peptides (e.g., serotonin, norepinephrine, dopamine, neuropeptide Y, and corticotropin-releasing hormone) were involved in the relationship between certain forms of mental distress (e.g., somatization) and the regulation of food intake or body weight [52,53]. Moreover, at a metabolic level, both high BMI and psychopathology have been associated with glucose intolerance and insulin resistance [54]. From a psychological point of view, the relationship linking psychopathological risk and obesity has been suggested to produce a vicious circle in which compromised physical function and obesity-related social stigmatization may result in higher levels of mental distress in obese individuals, while disordered eating (e.g., emotional eating) may foster obesity in subjects with poor mental health. It must be stated that the relationship between poor mental health and obesity is complex and remains unclear [55]. The literature has not thus far reached an agreement as to whether psychopathological symptoms may be considered as predisposing factors for the development of obesity or whether they should instead be understood as consequences of the disease. Some authors have even suggested a third hypothesis that recognizes the interaction between psychopathology and organic pathology, with no predominant role for either. Some authors have indeed found a causal effect [56] of psychopathological symptoms on BMI both in male and female obese individuals. However, they demonstrated different configurations of these symptoms for men and women. Our results are partially consistent with these studies, as somatization predicted BMI scores in both men and women (higher symptoms were correlated to higher BMIs). This finding might indicate that somatization is a “core” psychopathological symptom in obese subjects—regardless of their sex—that is capable of being a predictor for a higher BMI. The effect of men’s psychoticism on their BMI is consistent with Faith et al. [57], who posited that the influence of this variable on BMI was solely found in male obese subjects.

In women, attentional impulsivity had an effect on BMI. Further studies are needed to clarify why attentional impulsivity showed such a noticeable role in predicting obesity, especially considering that this role was not shown for any of the other subscales of the BIS. However, Hou et al. [58] suggested that attentional impulsivity could increase attention and susceptibility to highly palatable food cues, therefore triggering overeating (and consequently obesity). 

The present study has several strengths, and could therefore have both immediate and long-lasting positive consequences for the creation and application of treatment plans with obese patients. First, the vast majority of previous studies addressing the psychological profiles of obese subjects were based on the psychopathological assessment of states of depression and/or anxiety. However, apart from anxiety and depressive symptoms, obese people frequently show impulsivity, feelings of guilt for disordered eating, discouragement, hostility, obsessive-compulsive behaviors, and somatic complaints. Consequently, our research addressed a broader spectrum of psychopathological symptoms through a widely-used measure (the SCL-90). Second, our study considered the possible effect of gender in shaping the psychological profiles of obese patients and the influence of psychopathological risk on BMI, as has been highly recommended by several reputable authors [59,60]. Third, our study adjusted all results to account for all the confounding variables indicated by Di Pietro et al. [31], allowing for more robust findings. Finally, this study is based in the work of a multidisciplinary team composed of specialists in the medical and psychological fields, which collaborated to program interventions aimed to cause a desirable shift in the psychological status and lifestyle of patients and consequent weight loss. In Italy, the integration of multidisciplinary teams is infrequent, due to the limited resources available for health services that precludes effective collaboration between physicians and psychologists.

We are also aware that our study contains some limitations. We acknowledge the seminal work of Friedman and Brownell [50], which warned against further studies aimed at demonstrating a causal link between psychopathology and obesity. The wide heterogeneity in the etiology of obesity and the cross-sectional nature of our study combined with the relative paucity of the sample do not allow for firm conclusions about the causality of the relationship linking psychopathology and impulsivity to increased BMI in obese patients, nor do these issues permit generalizability of our results. However, it has been stated that, in clinical studies, sample size should not prevent drawing clinical conclusions, which are useful for the planning of programs for prevention and intervention methods that are more effective [61]. In addition, several studies [62] have implied the usefulness of cross-sectional studies even for assessing causal links between variables, and other authors have suggested that results from relatively small samples in clinical settings are still informative for the programming of assessment protocols and intervention plans [63].

## 5. Conclusions 

Obesity has reached the proportions of a global pandemic, with a worldwide prevalence of approximately 37% for men and 38% for women. The psychological assessment of obese patients represents a key point for proper diagnosis and treatment, as intervention plans to reduce weight should be primarily based on changing behavioral habits. 

This study focused on the psychological profiles of adult male and female obese patients, verifying the possible association of psychopathological risk and impulsivity with their BMI and perceived quality of life. While somatization, psychoticism and attention impulsivity were found to be associated with higher BMI, no effect of psychopathological symptoms was detected on the patients’ perceived quality of life. The capability of the subjects of this study to effectively report psychological suffering while being incapable of identifying the impact of obesity on their social and relational adjustment might be predicted by their poor metalizing capacities and should be evaluated in further studies.

Although in a cross-sectional design, these results might constitute a pivotal step forward to demonstrate a causal link between psychopathological symptoms and obesity in adults.

## Figures and Tables

**Table 1:** Mean and standard deviations of anthropometric and psychological measurements, differentiated on the basis of gender. BIS: Barratt Impulsiveness Scale; BMI: body mass index; ORWELL 97: Obesity-Related Well-Being questionnaire.

Measures		Men	Women	*p*
Anthropometric Measurements	Body weight	134.13 ± 25.97 kg	101.18 ± 20.46	0.000
	Body height	1.79 ± 0.06 m	1.64 ± 0.06 m	0.000
	Waist circumference	133.3 ± 15.3 cm	116.9 ± 14.8 cm	0.000
	BMI	42 ± 8.2 kg/m^2^	37.5 ± 6.9 kg/m^2^	0.020
Psychopathological Symptoms	Somatization	0.76 ± 0.53	1.3 ± 0.74	0.003
	Obsessive-compulsivity	0.69 ± 0.55	1.16 ± 0.83	0.015
	Interpersonal Sensitivity	0.56 ± 0.51	0.95 ± 0.92	0.046
	Depression	0.56 ± 0.46	1.2 ± 0.85	0.001
	Anxiety	0.41 ± 0.33	0.85 ± 0.61	0.002
	Hostility	0.51 ± 0.49	0.67 ± 0.72	0.361
	Phobic anxiety	0.14 ± 0.19	0.4 ± 0.55	0.027
	Paranoid ideation	0.63 ± 0.48	0.94 ± 0.89	0.123
	Psychoticism	0.24 ± 0.23	0.4 ± 0.5	0.138
	Global Severity Index	0.54 ± 0.33	0.93 ± 0.64	0.006
Impulsivity	Attention impulsivity	13.33 ± 2.3	14.5 ± 3.9	0.183
	Motor impulsivity	18.9 ± 3.8	20.11 ± 3.7	0.212
	Non-planning impulsivity	24.67 ± 4.07	25.8 ± 4.5	0.301
	BIS total score	55.9 ± 2.49	56.4 ± 3.4	0.129
Quality of Life	ORWELL 97	45.79 ± 17.97	50.09 ± 12.29	0.298

**Table 2:** *R*^2^, beta, *t*, and *p* values of regression analysis, conducted to evaluate the possible impact of men’s psychopathological symptoms and impulsivity (predictor variables) on their BMI and quality of life (dependent variables).

Psychological Subscales	BMI			QoL		
	Beta ^§^	*t*	*p*	Beta ^§^	*t*	*p*
Somatization	0.87	3	<0.05	−0.08	−0.26	Ns
Obsessive-Compulsivity	0.433	1.1	Ns	0.69	1.59	Ns
Interpersonal Sensitivity	−0.021	−0.07	Ns	−0.37	−1.1	Ns
Depression	0.015	0.04	Ns	0.58	1.32	Ns
Anxiety	−1.15	−2.14	Ns	−1.09	−1.84	Ns
Hostility	0.01	0.03	Ns	0.36	0.85	Ns
Phobic Anxiety	0.49	1.85	Ns	0.2	0.67	Ns
Paranoid Ideation	−0.15	−0.35	Ns	0.49	1.03	Ns
Psychoticism	0.629	2.43	<0.05	0.55	1.91	Ns
Attention Impulsivity	−0.47	−1.5	Ns	−0.44	−1.28	Ns
Motor Impulsivity	−0.65	−1.75	Ns	0.41	1.01	Ns
Non-Planning Impulsivity	0.47	1.69	Ns	−0.71	−2.33	Ns
*R*^2^	0.708			0.642		
Adjusted *R*^2^	0.690			0.652		

Note: § = Adjusted for smoking, alcohol use, concurrent medical illness and socioeconomic status. Ns, non-significant.

**Table 3:** *R*^2^, beta, *t*, and *p* values of regression analysis, conducted to evaluate the possible impact of women’s psychopathological symptoms and impulsivity (predictor variables) on the dependent variables BMI and quality of life (QoL).

Psychological Subscales	BMI			QoL		
	Beta ^§^	*t*	*p*	Beta ^§^	*t*	*p*
Somatization	0.46	2.33	<0.05	−0.11	−0.78	Ns
Obsessive-Compulsivity	−0.13	−0.45	Ns	−0.15	−0.73	Ns
Interpersonal Sensitivity	0.55	1.58	Ns	0.15	0.61	Ns
Depression	0.15	0.39	Ns	0.24	0.37	Ns
Anxiety	0.099	0.22	Ns	−0.29	−0.92	Ns
Hostility	0.39	1.42	Ns	0.25	1.3	Ns
Phobic Anxiety	−0.24	−0.898	Ns	0.33	1.84	Ns
Paranoid Ideation	0.11	0.29	Ns	−0.13	−0.48	Ns
Psychoticism	−0.074	−0.21	Ns	0.04	0.155	Ns
Attention Impulsivity	0.5	2.18	<0.05	0.03	0.52	Ns
Motor Impulsivity	−0.24	−1.27	Ns	0.34	0.86	Ns
Non-Planning Impulsivity	0.22	0.18	Ns	−0.28	−0.51	Ns
*R*^2^	0.419			0.723		
Adjusted *R*^2^	0.412			0.719		

Note: § = Adjusted for smoking, alcohol use, concurrent medical illness and socioeconomic status.
